# Supplementary figures and images for: Parental thermal conditions affect the brain activity response to alarm cue in larval zebrafish
Source: PeerJ. 2024 Oct 10;12:e18241. doi: 10.7717/peerj.18241 (PMC11471146; doi:10.7717/peerj.18241)

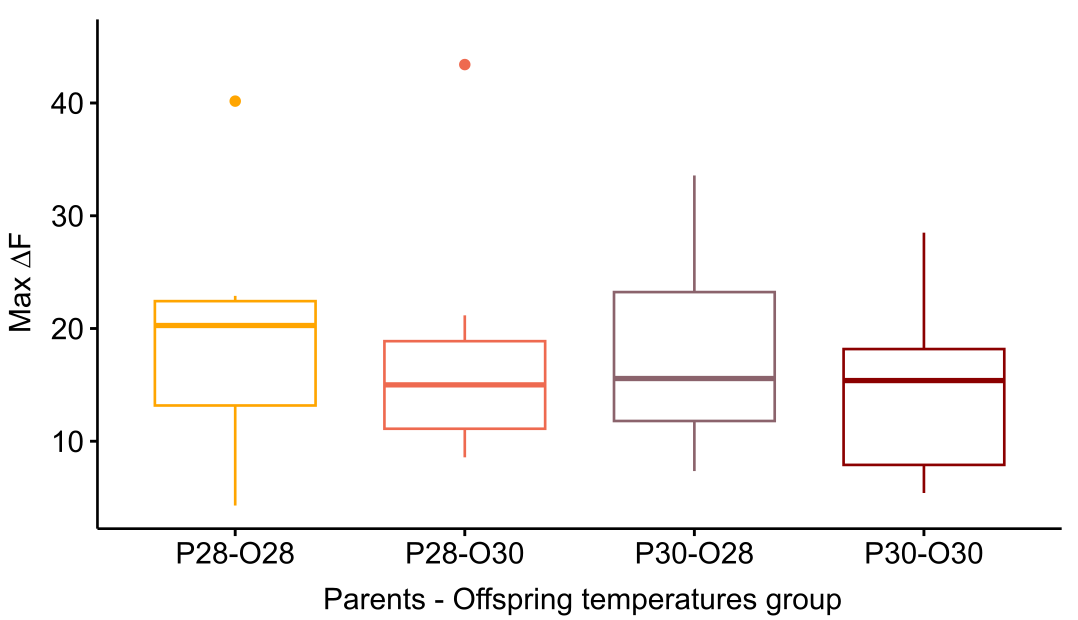

Supplement: Supplemental Information 1 — Parents that produced these offspring experienced either control (P28) or elevated temperature (P30) conditions; larvae were reared in either control (O28) or elevated temperature (O30) conditions [file peerj-12-18241-s001.png]

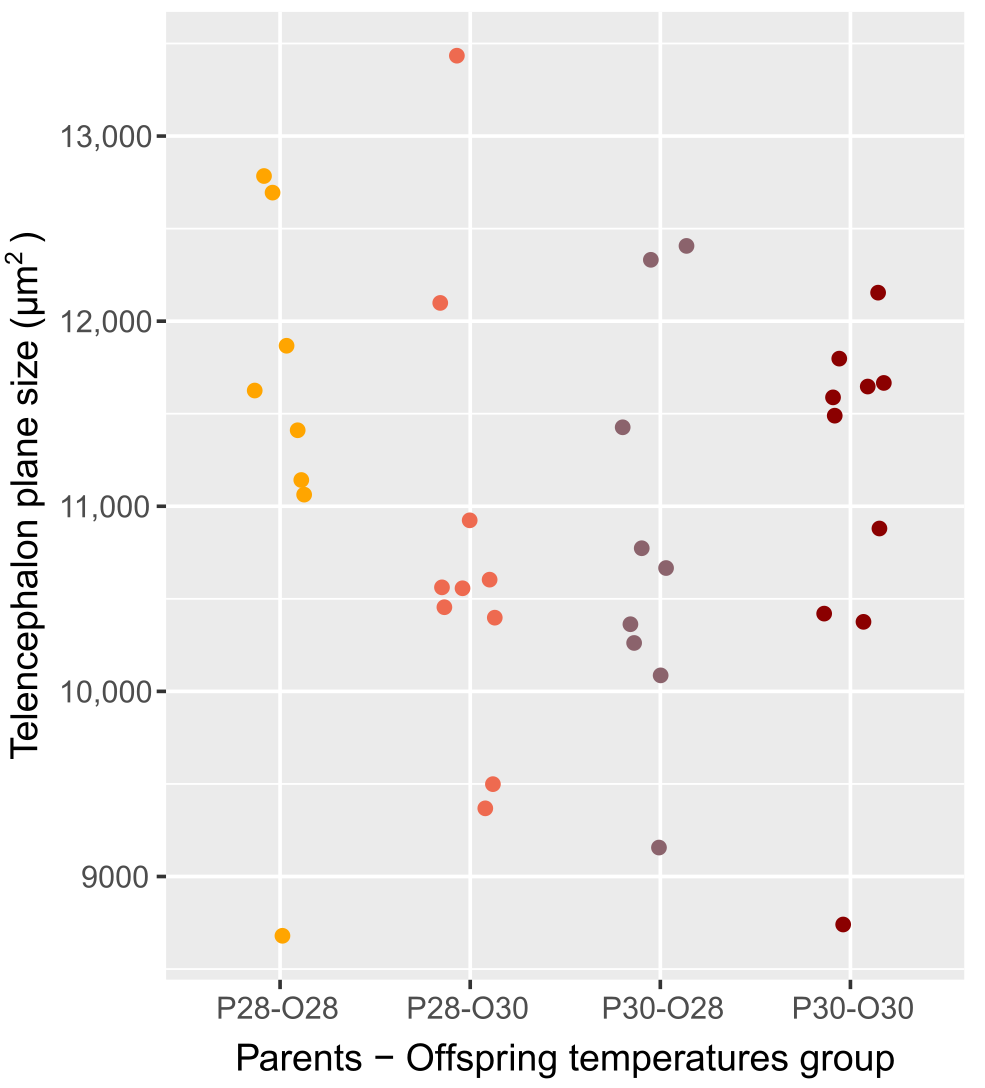

Supplement: Supplemental Information 2 — Parents that produced these offspring experienced either control (P28) or elevated temperature (P30) conditions; larvae were reared in either control (O28) or elevated temperature (O30) conditions [file peerj-12-18241-s002.png]
